# Endoscopic full-thickness resection: precision and efficacy in treating a giant duodenal stromal tumor

**DOI:** 10.1055/a-2671-0098

**Published:** 2025-08-14

**Authors:** Jiang Du, Yuan Gao, Lifan Zhang, Qiongying Zhang, Bing Hu, Yi Mou

**Affiliations:** 1 610464 Gastroenterology, Sichuan University West China Hospital School of Nursing, Chengdu, China; 2 34753 Gastroenterology and Hepatology/Medical Engineering Integration Laboratory of Digestive Endoscopy, Sichuan University West China Hospital, Chengdu, China

Duodenal stromal tumors, a rare subset of gastrointestinal stromal tumor (GIST), have traditionally been managed by surgical resection 1 ; however, owing to the complex anatomy of the duodenum, surgical approaches such as pancreaticoduodenectomy are associated with high morbidity and poor long-term outcomes 2 3 . Endoscopic full-thickness resection (EFTR) is emerging as a promising minimally invasive technique for treating gastrointestinal tumors 4 . Here, we present a case demonstrating the successful application of EFTR in treating a giant duodenal stromal tumor.

A 47-year-old woman presented with melena. Gastroscopy revealed a 2-cm submucosal lesion with surface ulceration in the duodenum ( Fig. 1 ). Pathologic examination of a biopsy suggested it was a GIST. Given the patient’s condition, endoscopic dissection was chosen as the treatment option ( Fig. 2 ; Video 1 ). First, a submucosal injection was carried out to facilitate the dissection. The tumor was then dissected layer by layer, and it was found that the tumor extended outside the lumen. An EFTR was performed until the tumor was completely resected. After that, the defect was closed with a double-layer purse-string suture. A nylon loop and titanium clips were used for the closure. Abdominal paracentesis was performed to evacuate intra-abdominal gas, and a nasoenteric tube was inserted for gastrointestinal decompression.

**Fig. 1:**
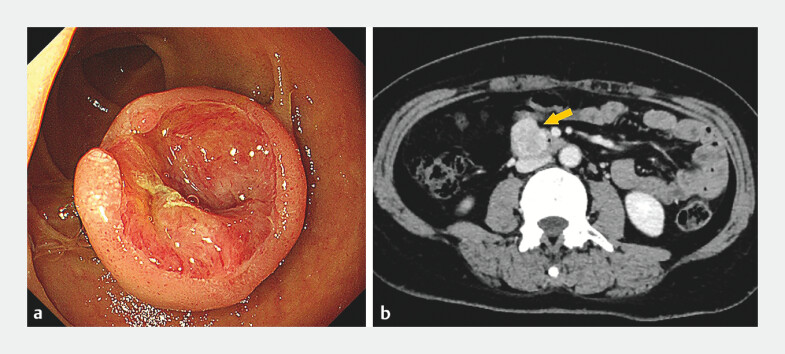
Preoperative examination of the duodenal lesion showing: **a** the appearance on gastroscopy with white light; **b** a contrast-enhanced computed tomography image suggestive of a gastrointestinal stromal tumor (yellow arrow).

**Fig. 2:**
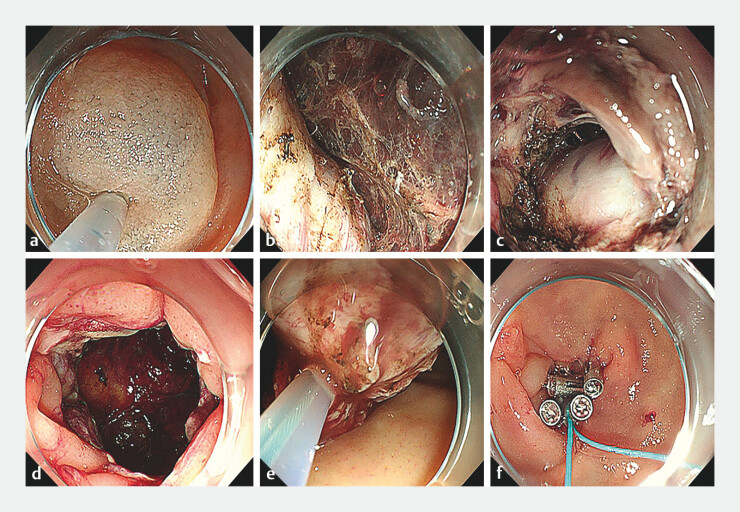
Endoscopic images during endoscopic full-thickness resection for a duodenal stromal tumor showing: **a** submucosal injection being performed; **b** layer by layer resection of the tumor; **c** extension of the tumor outside the lumen; **d** continued full-thickness incision until the tumor was completely dissected; **e** removal of the tumor using a snare; **f** closure of the defect with a double-layer purse-string suture.

Endoscopic full-thickness resection is performed to precisely and effectively resect a giant duodenal stromal tumor.Video 1

The resected specimen measured approximately 4.0 × 3.0 × 2.0 cm. Postoperative pathology confirmed it to be a GIST, with R0 resection ( Fig. 3 ). During a 2-year follow-up period, there were no complications, nor evidence of tumor recurrence or metastasis ( Fig. 4 ).

**Fig. 3:**
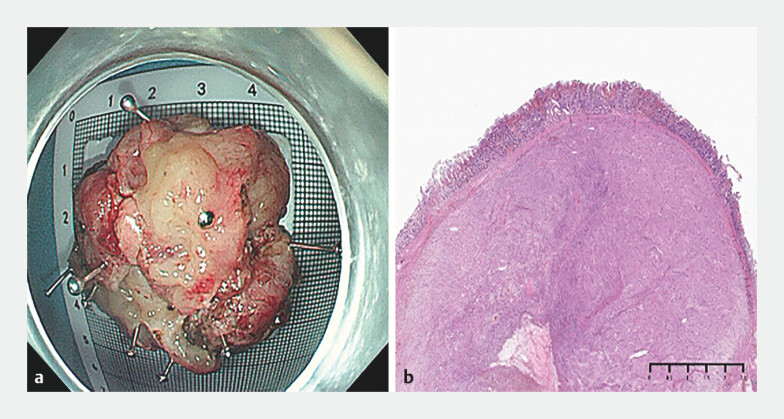
Appearance of the resected specimen, which was confirmed to be a gastrointestinal stromal tumor: **a** macroscopically; **b** histopathologically with hematoxylin and eosin (H&E) staining.

**Fig. 4:**
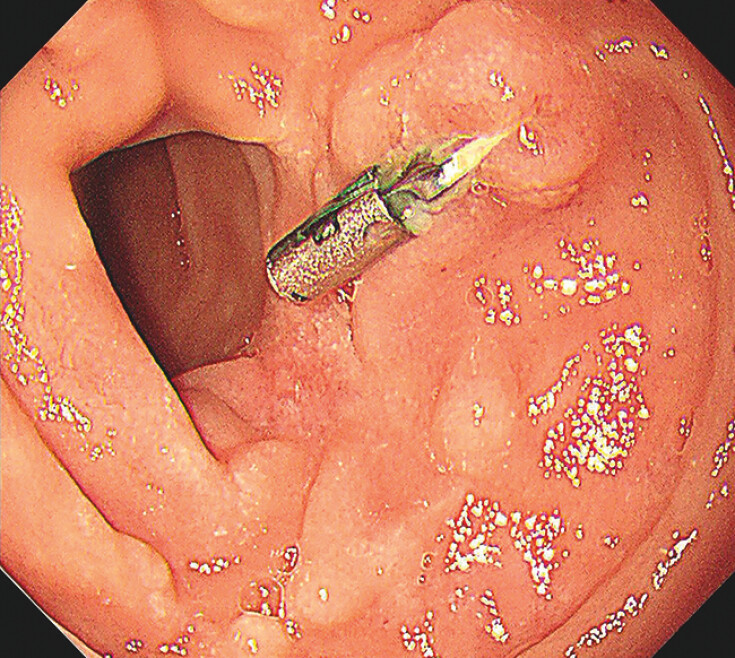
Appearance of the wound on follow-up gastroscopy 6 months after endoscopic full-thickness resection.

EFTR offers a less invasive alternative to traditional surgery for duodenal stromal tumors, particularly addressing the high morbidity associated with pancreaticoduodenectomy. This case highlights the potential of EFTR in treating duodenal stromal tumors, providing a new treatment option with reduced trauma and better prognosis.

Endoscopy_UCTN_Code_TTT_1AO_2AG_3AZ

## Notes

Endoscopy E-Videos https://eref.thieme.de/e-videos
*E-Videos* is an open access online section of the journal *Endoscopy* , reporting on interesting cases and new techniques in gastroenterological endoscopy. All papers include a high-quality video and are published with a Creative Commons CC-BY license. Endoscopy E-Videos qualify for HINARI discounts and waivers and eligibility is automatically checked during the submission process. We grant 100% waivers to articles whose corresponding authors are based in Group A countries and 50% waivers to those who are based in Group B countries as classified by Research4Life (see: https://www.research4life.org/access/eligibility/ ). This section has its own submission website at https://mc.manuscriptcentral.com/e-videos .
